# Essential Oils and Antifungal Activity

**DOI:** 10.3390/ph10040086

**Published:** 2017-11-02

**Authors:** Filomena Nazzaro, Florinda Fratianni, Raffaele Coppola, Vincenzo De Feo

**Affiliations:** 1 Institute of Food Science-National Research Council (CNR-ISA), Via Roma 64, 83100 Avellino, Italy; fratianni@isa.cnr.it; 2 DiAAA, Molise University, Via de Sanctis s.n., 86100 Campobasso, Italy; coppola@unimol.it; 3 Department of Pharmacy, University of Salerno, Via Giovanni Paolo II, 132, 84084 Fisciano (SA), Italy; defeo@unisa.it

**Keywords:** essential oils, antifungal, biofilm, mycotoxins, quorum sensing, human health

## Abstract

Since ancient times, folk medicine and agro-food science have benefitted from the use of plant derivatives, such as essential oils, to combat different diseases, as well as to preserve food. In Nature, essential oils play a fundamental role in protecting the plant from biotic and abiotic attacks to which it may be subjected. Many researchers have analyzed in detail the modes of action of essential oils and most of their components. The purpose of this brief review is to describe the properties of essential oils, principally as antifungal agents, and their role in blocking cell communication mechanisms, fungal biofilm formation, and mycotoxin production.

## 1. Essential Oils

There is an increasing demand to reduce the use of chemicals as antimicrobial agents in the field of nutrition and to combat various infections due to increasingly aggressive and increasingly endogenous microorganisms that are resistant to the use of synthetic antimicrobials. In this direction, substances derived from plants, such as hydro-alcoholic extracts or essential oils, can certainly play a fundamental role. The versatility of such substances is enormous; the same plant can provide a pool of substances with a very broad spectrum of action due to their different chemical structure. Furthermore, the hypersensitivity and toxicity to the drugs, because of their improper and excessive application, represent some of the major problems of the conventional medicine consequences of the presently excessive use of synthetic antimicrobials. Public awareness has therefore generated interest in the application of natural substances already used throughout the ages for the treatment of certain diseases transmitted by organisms. The use of essential oils is common, even since the earliest civilizations, firstly in the East and the Middle East, then in North Africa and Europe [1]. The term “Essential Oil” (EO) was coined in the 16th century by the Swiss reformer of medicine, Paracelsus von Hohenheim. Plant EOs are usually complex mixtures of natural compounds, both polar and non-polar [1,2]. Well-known for their antiseptic and medicinal properties (analgesic, sedative, anti-inflammatory, spasmolytic, local anesthetic, anti-carcinogenic), they are also used in embalmment, and, due to their antimicrobial and antioxidant activity, as natural additives in foods and food products [3,4,5,6]. The International Organization for Standardization (ISO) (ISO/D1S9235.2) defines an essential oil as a product made by distillation with either water or steam or by mechanical processing or by dry distillation of natural materials. They appear as liquid, volatile, limpid and colored mixtures of several aromatic compounds. EOs are obtained from all plant parts, mainly from herbs and spices, although at present new sources of EOs are examined, for example from food and vegetal wastes [7,8]. About 3000 EOs are known, 300 of which are commercially important, mainly used in the flavors and fragrances market [6]. In nature, EOs play an important role in the protection of plants against undesirable enemies. As for other plant metabolites, the role of EOs is the protection of the plant organism against some pathogenic microorganisms, the exertion of a repel action towards insects that act as plague vectors, and the reduction of the appetite of some herbivores (by inducing unpleasant taste to the plant). On the other hand, they also may attract some insects to promote the dispersion of pollens and seeds. Thus, EOs can play a role in mediating the interactions of plants with the environment [4]. The main categories of compounds are terpenes and terpenoids; rarely nitrogen- and sulphur-containing compounds, coumarins and homologues of phenylpropanoids can also be found [9,10]. Terpenes are a large class of naturally occurring hydrocarbons, deriving from the isoprene unit (C_5_H_8_), with various chemical features and biological properties. They are synthesized in the cytoplasm of plant cells through the pathway of mevalonic acid starting from acetyl CoA. Monoterpenes (C_10_H_16_) and sesquiterpenes (C_15_H_24_) are generally the principal terpenes, but longer chains such as diterpenes (C_20_H_32_), triterpenes (C_30_H_40_), etc., also exist. Examples of terpenes include *p*-cymene, limonene, terpinene, sabinene and α- and β-pinene. Monoterpenes are constructed from the coupling of two isoprene units. They constitute 90% of the essential oils and allow a great variety of structures. Some of the major compounds include monoterpene hydrocarbons (such as limonene, *p*-cymene, α-pinene, and α-terpinene), and oxygenated monoterpenes (such as carvacrol, thymol and camphor). Sesquiterpenes are formed from the assembly of three isoprene units. The extension of the chain increases the number of cyclizations permitting a great variety of structures. The structure and function of sesquiterpenes are comparable to those of the monoterpenes. Terpenoids are compounds related to terpenes, with some oxygen functionality or some rearrangement. Thymol, carvacrol, linalyl acetate, linalool, piperitone, citronellal, geraniol and menthol are considered as the most known terpenoids. Within each group, molecules can be simply unsatured hydrocarbons, or contain functional groups; in this last case, they are acids, alcohols, aldehydes, ketones, esters [10,11]. Chemical composition of plant essential oils differ among species; it is affected by factors including the geographical location, environment, the stage of maturity and method of extraction. This chemical difference is directly correlated to differences in biological activities [12,13]. EOs and their components have a variety of targets, particularly the membrane and cytoplasm, and in certain situations, they completely alter the morphology of the cells [13]. Both humans and plants are susceptible to fungal infections by pathogenic fungi and some synthetic fungicides are known to be effective in their control. However, the use of synthetic fungicides is limited by the emergence of resistant fungus strains and some fungicides possess considerable toxicity. Moreover, there is a growing public concern over the increased health and environmental hazard associated with synthetic molecules. For this reason, alternative, safe and natural methods to develop new antifungal agents are actively studied [11]. Recently, there has been a great interest in using essential oils as possible natural substitutes for conventional synthetic fungicides [14].

## 2. Antifungal Activity

Fungal infections are caused by eukaryotic organisms, and it is therefore more difficult to ascertain their presence and apply the appropriate therapeutic treatment compared to bacterial infections. The cell wall of fungi may be considered as the prime target for selectively toxic antifungal agents because of its chitin structure, which is absent in human cells. Chemical treatments are largely effective, but resistant strains and intrinsically resistant species can be developed. The onset and severity of the fungal infection depends on the inoculum charge, the host’s immunological state and resistance. EOs can represent one of the most promising natural products for fungal inhibition [15,16]. In fact, many kinds of EOs obtained from different plants or herbs exhibited intense antifungal properties [4,15,17,18,19]. EOs, like the other phytochemicals, could attenuate the microbial growth and biofilm development through specific mechanisms [10]. This aspect is of particular value: It is well known that, in addition to a certain growth threshold value, microorganisms trigger a certain mechanism leading to the synthesis and production of molecules, microbial communication signals, and the development of specific pathogenicity parameters, such as the development of biofilms. Due to their extensive antimicrobial properties, many essential oils could be used for the control of microbial spoilage, the preservation of food quality and safety, and the prolongation of their shelf life [20]. Essential oils are classified as “Generally Recognised as Safe” (GRAS) by the Food and Drugs Administration (FDA), thus they are not harmful and, due to their natural origin, are more widely accepted by consumers than “synthetic” agents [21]. The antimicrobial or antifungal activity of essential oil might be caused by the properties of terpenes/terpenoids, that—due to their highly lipophilic nature and low molecular weight—are capable of disrupting the cell membrane, causing cell death or inhibiting the sporulation and germination of food spoilage fungi. Therefore, several in vitro tests indicate that terpenes/terpenoids show ineffective antimicrobial activity when used as singular compounds compared to the whole EO [22,23]. All the activities exhibited by essential oils and/or their component, which will be discussed in this review, are resumed in Table 1. 

**Figure 1:**
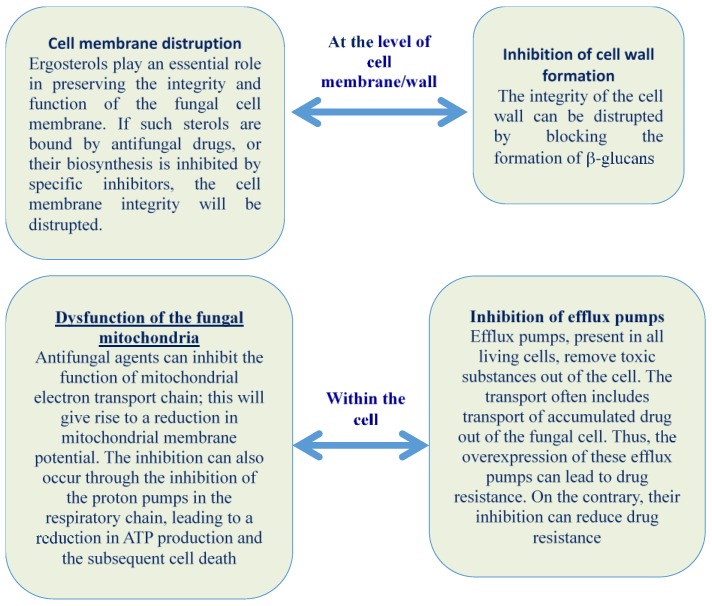
EOs action against fungi.

According to Freiesleben and Jager [88], the antifungal agents can deactivate the fungus by disrupting the structure and function of membranes or organelles of fungal cell and/or inhibiting the nuclear material or protein synthesis (Figure 1).

Some of the mechanisms of essential oils and/or their components are described below.

### 2.1. Cell Membrane Disruption, Alteration, and Inhibition of Cell Wall Formation 

The fungal cell wall plays an important role in the growth and viability of fungi; the three major structural elements, glucan, chitin, and mannan, are generally considered therapeutic targets. Chitin, a long linear homopolymer of β-1,4-linked *N*-acetylglucosamine (GlcNAc), is synthesized in a reaction catalysed by chitin synthase. Chitin is indispensable for the construction of the cell wall, and therefore, for fungal survival. The inhibition of chitin polymerization may affect cell wall maturation, septum formation, and bud ring formation, by damaging cell division and cell growth [24]. Trans-anethole, a major component of anise oil demonstrated antifungal activity against the filamentous fungus, *Mucor mucedo* IFO 7684, accompanied by hyphal morphological changes such as swollen hyphae at the tips [25]. Anethole dose-dependently inhibited chitin synthase (CHS) activity in permeabilised hyphae. The essential oil from *Citrus sinensis* epicarp (composed on limonene at 84.2%) is capable of inhibiting the growth of *Aspergillus niger*; it also leads to irreversible deleterious morphological alterations (in particular the loss of cytoplasm in fungal hyphae, and budding of hyphal tip [26]. Iscan et al. [27] revealed the extensive fungal cell wall and cytoplasmic membrane damage after exposure to thymoquinone, the major component of the black cumin seed essential oil. Also tea tree EO and its components are capable to alter both permeability and membrane fluidity of *Candida albicans* by altering membrane properties and compromising membrane-associated functions. The action of such EO causes also a thinning and distortion of the hyphal wall, and subsequent cell wall disruption, and a final transformation to flatten and empty hyphal tips bifurcated into bud like structures [28]. Some EOs or their components can act at singular or multiple level. The EO of *Litsea cubeba* and its component citral are capable to exhibit antifungal activity against *Fusarium moniliforme*, *F. solani*, *Alternaria alternata* and *A. niger*, through damage of their cell wall and cell membrane to various degrees, cytoplasm leakage and partially by inhibiting DNA, RNA, protein and peptidoglycan biosynthesis; in addition, they can inhibit the ergosterol biosynthesis by *C. albicans*. Similar effect is exhibited by *Coriaria nepalensis* and *Coriandrum sativum*, both by the whole essential oils and by some of their components, which act through the disruption of normal sterol biosynthetic pathways, leading to a reduction in biosynthesis of ergosterol a compound similar to cholesterol, present in the fungal cell membrane. Absence or reduced presence of ergosterol in fungal membranes results in osmotic and metabolic instability of the fungal cell, compromising reproduction and infectious activity [29,30,31,32].

### 2.2. Dysfunction of the Fungal Mitochondria

Some essential oils can affect the mitochondrial effectiveness by inhibiting the action of mitochondrial dehydrogenases, involved in ATP biosynthesis, such as lactate dehydrogenase, malate dehydrogenase and succinate dehydrogenase. Chen et al. [33] showed that the EO of *Anethum graveolens* was capable to act as antifungal agent also through disturbance of the citric acid cycle and inhibition of ATP synthesis in the mitochondria of *C. albicans*. In another situation, EOs of *Origanum compactum*, *Artemisia herba alba* and *Cinnamomum camphora* showed an increase of the number of cytoplasmic petite mutations, i.e., mitochondrial damage, in *Saccharomyces cerevisiae* [34]. Haque et al. [35] indicated that terpenoids could play a key role in diminishing the mitochondrial content, which gives rise to an altered level of reactive oxygen species (ROS) and ATP generation.

### 2.3. Inhibition of Efflux Pumps 

The fungal plasma membrane H^+^-ATPase plays an important role in the physiology of the fungal cell by supporting the large transmembrane electrochemical proton gradient across cell membrane necessary for nutrient uptake. The H^+^-ATPase also regulates intracellular pH and fungal cell growth; furthermore, it is involved in fungal pathogenicity through its effects on dimorphism, nutrient uptake, and medium acidification [35,36,37]. Inhibition of H^+^-ATPase leads to intracellular acidification and cell death. Ahmad et al. [38] showed that eugenol and thymol are excellent fungicidal agents, including those resistant to azoles, and the action is probably located at membrane level. Both compounds inhibit H^+^-ATPase activity. Thymol and carvacrol, the principal chemical components of thyme oil exhibited a synergistic antifungal effect with azole antimycotic fluconazole by inhibiting the over-expression of efflux-pump genes CDR1 and MDR1 in *C. albicans*. Both monoterpenes inhibited efflux by 70–90%, showing their high potency to block drug transporter pumps.

### 2.4. ROS Production 

Bacterial nitric oxide synthases (bNOS) synthesize nitric oxide (NO) from arginine. NO generated by bNOS increases the resistance of bacteria to a broad spectrum of antibiotics, allowing the bacteria to survive and share habitats with antibiotic-producing microorganisms. NO-mediated resistance is achieved through both the chemical modification of toxic compounds and the alleviation of the oxidative stress imposed by many antibiotics. Thus, the inhibition of NOS activity may increase the effectiveness of antimicrobial therapy [39,40]. Few reports have mentioned that endogenous NO in fungi mediated the cell death in response to the chemical stresses. Fungi are capable of synthesizing NO [41,42]. NO is also involved in the protection of mycelia of edible fungi from heat stress-induced oxidative damage [43]. Recent studies show that even if many antibiotics have different targets in bacteria, the lethal actions are common by the generation of reactive oxygen species (ROS), which revealed to play important roles in cell death also among species from bacteria and fungi [44]. EOs oil can reduce the level of nitric oxide, and limit H_2_O_2_ production and NO synthase, demonstrating a potential in the therapy of oxidative damage [45,46]. Thymol, one of the most famous components of thyme, exhibited efficient fungicidal activity against *Aspergillus flavus*, a fact that reveals its great potential as drugs for the medical treatment of aspergillosis disease. Therefore, thymol was supposed to be used on crops to decrease the infection of *A. flavus*, through the ROS scavengers [41].

Many studies reported that the constituents of essential oils [47] could act synergistic or antagonistic [48]. The combination of some particular oils can increase fungistatic activity. This feature would have the advantage in pre- and post-harvest protection of crops, and in extending the shelf life of packaged foods, because pathogens cannot easily acquire resistance to multiple components of two or more EOs. The antifungal activity of terpenoids is generally related to their functional groups, i.e., to the hydroxyl group of phenolic terpenoids. Carvacrol and thymol, produced from *p*-cymene, exhibit an important antifungal effect; they cause damage in the cell membrane by interacting with sterols and in particular with ergosterol. When tested alone, carvacrol, present in the essential oils of some oregano and thyme chemotypes, shows strong antifungal activity [49,50]. It is possible that carvacrol is capable to bind to the sterols of the fungal membrane, a fact that results in damage and, consequently, death of the fungus [51]. Thymol is structurally analogous to carvacrol, but the locations of the hydroxyl groups differ between the two molecules. However, these differences do not affect their activities; like eugenol, they act by causing damage in the cell membrane and interacting with ergosterol [52,53,54,55]. Thymol affects mycelium morphology, with changes in the localization of chitin within the hyphae. Similar mechanism was observed also in other monoterpenes, such as linalool, which acts also by interfering with fungal stability and biofilm formation. The structure and function of the plasma membrane of fungal cells is an essential key for the survival of these microorganisms; every important alteration or modification, which involves the synthesis or maintenance of the cell membrane can result in damages and, consequently, cause the death of the fungus. The antifungal activity of EOs and their components, as well as their capability to block the toxins production, can show different level of potency, depending on their structure. The monoterpenoid citronellal is a volatile constituent present in *Citrus* and other plant EOs with a well-known antifungal activity [56,57,58,59]. Zore et al. [60] observed that it could inhibit the growth of *C. albicans* by affecting membrane integrity and arresting cell cycle. Cinnamaldehyde is an active inhibitor of bacterial, yeasts, and filamentous moulds growth. It exerts its action through the inhibition of ATPases activity, cell wall biosynthesis, and alteration of the membrane structure and integrity. Consequently, cinnamaldehyde and its derivatives possess potential antifungal activity against several fungal isolates [61,62,63,64,65,66].

EOs components could act as antifungal agents due to their accumulation in the lipophilic hydrocarbon molecules of the cell lipid bi-layer; such action also allows the easier transfer of other EOs constituents to the inner of the cell. The different activity may be explained by the variety in water solubility and lipophilic properties of the EOs. For instance, cinnamon that contains linalool exhibits higher antifungal activity compared to basil and peppermint that contain methyl chavicol and menthol as their main components [67]. Specific functional groups can interfere with the membrane-associated enzyme proteins and affect inhibitory processes of EOs [67]. Some EOs (i.e., cinnamon leaf, cinnamon bark, lemon, eucalyptus, cedar wood, white grapefruit, pink grapefruit, mint, thyme, and rosemary) exhibited distinct activity not only in inhibiting the mycelia growth [68]. Such action is mainly due to the synergistic action of the molecules that are present in the respective EOs. The oxygenated sesquiterpenes and sesquiterpene hydrocarbons also exhibit antifungal activity and, in some cases, can be considered even an alternative antifungal material basis in respect to the whole EO, since they have a high efficiency and low drug fungal resistance. Some deoxyglucosides of carvacrol, thymol, menthol, fenchol, borneol, *S*-(–)-perillyl alcohol and citronellal generally exhibited remarkable in vitro antifungal properties against *Aspergillus*, such as *A. flavus*, *A. ochraceus* and *Fusarium*, for example *F. oxysporum* [69]. Citronellal, a typical terpenoid of plant EOs, was capable of inhibiting the mycelial growth and spores germination of *Penicllium digitatum* in a dose-dependent manner, by deteriorating the plasma membrane of *P. digitatum* spores and leading to a higher extracellular conductivity and release of cell constituents. Moreover, in vivo results demonstrated that treatment with citronellal effectively reduced the incidence of green mould after 5 days of storage at 25 ± 2 °C, suggesting that the plasma deterioration mechanism contributed to the antifungal activity of citronellal against *P. digitatum* [70].

## 3. Essential Oils and Biofilms

**Figure 2:**
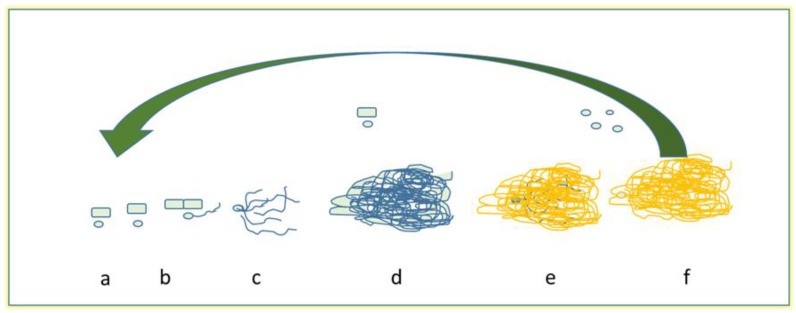
Model of biofilm development in filamentous fungi: (**a**) adsorption, (**b**) active attachment, (**c**) the first formation of microcolony through the process of germination and/or the development of a monolayer, (**d**) the development of mycelia, (**e**) the maturation of fungal biofilm, and finally (**f**) dispersion of conidia and/or and/or arthroconidia (modified from [77,89]).

In their natural environments, most of bacteria and fungi change from a planktonic to a sessile state forming the so-called biofilms [71]. Biofilms are sessile microbial and fungal communities that are strongly attached to surfaces and to each other; in such phase, they are protected by a polymeric extracellular matrix (ECM), constituted primarily of polysaccharides [72,73]. Biofilm formation has been studied mainly in bacteria and, among fungi, in the pathogenic yeast *C. albicans* or *A. fumigatus* [74,75]. Detailed gene expression profiling comparisons, conducted in both *C. albicans* and *A. fumigatus*, revealed substantial differences in gene expression between biofilm and planktonic cells, suggesting biofilm formation to be a highly regulated process. Due to this situation, cells of sessile communities show improved resistance against the external environment, and different phenotypes of planktonic or free cells are associated with the persistence of infections. The formation of fungal biofilm is of particular importance, for example, in medical field: in fact, pathogenic fungi, especially yeasts, can also adhere to surfaces such as catheters and prostheses, and can gain access to blood circulation, and they reach the internal organs of patients. Such development can also lead to a high mortality rate [74,76,77]. Biofilm cell communities show more resistance than planktonic cells to antifungal drugs. Contributing factors include, apart from biofilm structural complexity, the metabolic heterogeneity intrinsic to biofilm, the presence of the ECM, and a biofilm-associated up-regulation of efflux pump genes [78]. Nutrients, quorum-sensing molecules, and surface contact are contributory factors. Biofilm-associated resistance mechanisms include drug binding by ECM and production of persister cells [79], the latter representing only a fraction of the population, and probably reflecting its metabolic heterogeneity. These mechanisms may pertain to other fungi as well. In *Candida*, biofilms are composed of dense layers of yeast blastopores, hyphal, pseudohyphae, and ECM, which depends mainly on type of species [81,82,83]. The emerging fungal pathogen *Trichoderma asahii* forms biofilms comprised of yeast and hyphal cells embedded in matrix [84], as that of *Coccidioides immitis* [85]. *Cryptococcus neoformans* forms biofilms consisting of yeast cells on many abiotic substrates [86], and shed capsular polysaccharide forms the ECM. Although *C. neoformans* forms hyphae in the course of mating, no hyphae have been observed in *C. neoformans* biofilms to date. Similarly, *Pneumocystis species* do not produce hyphal structures as part of their biofilms [87]. Thus, hyphal formation is not a uniform feature of fungal biofilms. Costa-Orlandi et al. [72] described a general development of the filamentous fungi biofilms (Figure 2). 

The steps generally include: (a) propagule adsorption, which involves contact of spores, hyphal fragments, or sporangia to a surface; (b) active adhesion, in which spores secrete proteins adhesins and other reproductive structures during germination; (c) first microcolony formation, regarding the elongation and hyphal branching, that forms a monolayer with the production of extracellular matrix; (d) the formation of another microcolony (also called initial maturation), in which we could see a 3-D compact hyphae networks, covering by an extracellular matrix, and formation of water channels; (e) final maturation: in this case, fruiting bodies and other survivor structures are formed varying on the fungi; lastly, (f) the dispersion or planktonic phase, in which conidia and/or hyphae fragments are released, ready to start a new cycle. In this last phase, intact pieces of biofilm can be released, and carried to a new site of infection. During the biofilm formation, filamentous fungi secrete also other proteins, such as the small proteins hydrophobins, which are certainly involved in the process of hyphal adhesion to the hydrophobic surfaces and probably are also involved in biofilm formation [90].

Biofilm cells show both phenotypic and genotypic differences when compared to the planktonic ones. Gene expression of different pathways, for example, can be up or down regulated in the biofilm cells in contrast with the planktonic form, and more than 3000 differentially regulated genes have been identified under the two conditions [77,89]. Some of these genes reveal antifungal resistance or up regulation of secondary metabolite pathways [76]. Genetic exchange is a feature of bacterial biofilms, mediated in part by extracellular DNA. The main mechanism of biofilm-associated genetic exchange involves mating and cell fusion. Most biofilm studies have been conducted with non-mating *a*/*α* cells, but biofilm formation of the mating-capable cell types, *a*/*a* and α/α, has revealed a unique regulatory pathway intimately tied to pheromone signaling. The human fungal pathogen *C. albicans*, responsible for several nosocomial infections, is a quite extraordinary example of this can occur. Depending upon the configuration of the mating type locus (*a*/*α* versus *a*/*a* or *α*/*α*), *C. albicans* forms alternative biofilms that appear similar morphologically, but exhibit significantly different characteristics regulated by distinctly different signal transduction pathways. Biofilms formed by a/α cells are impermeable to molecules in the size range of 300 Da to 140 kDa, can be weakly penetrated by human polymorphonuclear leukocytes (PMNs), and show resistance to antifungals. On the contrary, *a*/*a* or *α*/*α* biofilms are permeable to molecules in this size range, are readily penetrated by PMNs, and are susceptible to antifungals. In order to mate, *C. albicans* must go through a switch from the white to opaque cell type. Upon switching, *α*/*α* opaque cells release a mating pheromone that induces a mating response in *a*/*a* opaque cells and vice versa. Pheromone release also induces an adhesive phenotype among the mating-incompetent *a*/*a* white cells, leading to mixed biofilm formation and ultimately mating [91,92].

Thus, *Candida* is the object of intense study, due to its phenotypic adaptation within the biofilm [93,94], and new synthetic drugs and derivatives are developed and studied to combat its infection. However, such molecules can also have undesirable effects on human health [95,96,97]. In addition, sometimes the action of these antifungals may be partially succeeded, due to an incomplete penetration and chemical reaction into biofilm matrix and the extracellular polymeric material. As in bacteria, the increasing resistance of fungi towards these antifungal compounds led to the search of new natural therapeutic alternatives deriving from plants, such as the essential oils. This research is also devoted to the discovery of new and effective natural products with antifungal activity and low cytotoxicity. Use of natural molecules has encouraged the examination of anti-biofilm action, since these molecules less likely induce resistant phenotypes, and their utilization is supported by a positive public perception of safer and eco-friendly options [98]. Tea tree oil, for example, has long been associated with antimicrobial and antifungal properties. It has direct activity against *C. albicans* biofilms as well as in inhibiting their formation [99,100]. Different EOs, such as those extracted by *C. sativum* [101] and *Ocimum americanum* [102], demonstrated an interesting activity as biofilm inhibitors in *C. albicans*. Other natural molecules, such as cinnamon oil, linalool, and xanthorrizol, appeared to possess biofilm inhibition properties [103,104,105]. Eugenol displayed in vitro activity against *C. albicans* cells within biofilms, when minimal inhibitory concentration (MIC_50_) for sessile cells was 500 mg/L, a value two-fold higher compared to the 48-h MIC_50_ of eugenol for planktonic *C. albicans* cells [106]. Essential oils are effective also in the treatment of polymicrobial biofilms, composed of bacteria and fungi, usually found in patients affected by chronic infections. Argawal et al. [107] demonstrated that peppermint, eucalyptus, ginger grass and clove EOs not only are capable of acting as potent antifungal agents against *C. albicans* and its biofilm, but especially eucalyptus oil exhibits a potentially superior antifungal agent compared to the conventional drug fluconazole. Further observation of the biofilm ultrastructure by Scanning Electronic Microscopy (SEM) revealed that damage to the biofilm constituents, despite the relative minimal diffusion, was due to the presence of eucalyptus and peppermint EOs that exerted a metabolic interference in *Candida* biofilm. Accordingly, the reduction of fungal biofilm by such EOs could be explained based on the presence of some active components that restrict biofilm development, such as 1,8-cineole, limonene and linalool, geranial, germacrene-D and menthol, components with a well-known antifungal activity. Indeed, the effective concentration of EOs is within the toxicity range for the mammalian cells [108], thus, such EOs have also low environmental and health impact. Furthermore, *Rosmarinus officinalis* EO when coated to nanoparticles strongly inhibited the adherence ability and biofilm development of *C. albicans* and *C. tropicalis* to the catheter surface, as shown by viable cell counts and confocal laser scanning microscopy examination [109]. Due to the important implications of *Candida* spp. in human pathogenesis, especially in the emergence of antifungal tolerance/resistance, the research conducted with free or coated shell based on essential oil could be of great interest for the biomedical field, opening new directions for the design of film-coated surfaces with anti-biofilm properties [110]. Thyme EO caused a “moulds” change to the form of vegetative hyphae in *Alternaria*, *Aureobasidium* and *Penicillium* by influencing also the biofilm formation [111]. Recently Khan and Ahmad [112] reported antibiofilm activity by oils of *Cymbopogon citratus* and *Syzygium aromaticum* against some drug resistant strains of *C. albicans*. Light and scanning electron microscopic studies ascertained the deformity of three-dimensional structures of biofilms formed in the presence of sub-MICs of oils. Thus, the cell membranes appeared to be the target site of compounds in biofilm cells as displayed by SEM observations. The atomic force microscopy (AFM) observation is useful in the imaging analysis of the fungal biofilm morphology on solid surfaces, both in the dried and hydrated states. Many researchers studied the biofilm structure of yeast and filamentous fungi using AFM to demonstrate the effectiveness of anti-biofilm agents and morphogenetic variations compared to the planktonic growth of cells. Observations made under SEM and AFM by Tyagi and Malik [109] on *Candida* cells and biofilm treated with the *C. citratus* oil led the researchers to differentiate the effect of liquid and vapour phase of oils on fungal biofilm cells and biofilm structure. The AFM picture of untreated cells showed cluster of *C. albicans* cells in intact shape. In the lemongrass essential oil treated cells, cells lost their original shape, and appeared shrunken and partially deformed. Although length and width seemed not to significantly affected, cells appeared relatively flattened indeed. The height of lemongrass essential oil treated and lemon grass essential oil vapour treated cells was found to be 40% and 90% less compared to the untreated cells (350 nm, 150 nm and 37.5 nm, respectively). AFM displayed also significant differences in the *Z*-axis value of the 3-D structure of the fungal cell. Results clearly demonstrated that the vapour treatment not only perceptibly altered the cell dimensions and the overall morphology, but also had a great impact on the cell surface properties. De Toledo et al. [113] demonstrated an effective anti-biofilm activity of EO of *C. nardus* against *C. albicans*, where transition of *C. albicans* from yeast to the hyphal form was inhibited. Microscopic observation of EO-treated fungal cells revealed an absence of filamentous hyphal form at concentrations ranging from 1000 μg/mL to 15 μg/mL. *C. nardus*, in particular, demonstrated important antifungal properties against the growth and biofilm formation of *Candida* yeasts, and MIC values generally ranged from 250 μg/mL to 1000 μg/mL. The EO inhibited hyphal formation of *C. albicans* strains at concentrations ranging from 15.8 μg/mL to 1000 μg/mL (depending also by the stage of biofilm maturity). The EO of *Laurus nobilis*, a plant native to the Mediterranean region belonging to the *Laureaceae* family is considered as a rich source of bioactive compounds [114]. *L. nobilis* EO demonstrated antifungal properties against *Candida* spp. biofilm adhesion and formation. MIC values of the EO increased in the presence of sorbitol as an osmotic protector, suggesting that it might affect *C. albicans* cell wall biosynthesis. This EO showed affinity for ergosterol, which could be involved in the imbalance and the ionic permeability of the cell membrane, but also in mature biofilm formation, activities similar to that exhibited by monodrug nystatin. *L. nobilis* EO even at concentrations from 1000 μg/mL significantly inhibited the adhesion of *C. albicans* much more effectively than nystatin [115]. This result is of particular meaning; in fact, the inhibition of yeast cell adhesion can be a target for disrupting the early stages of biofilm formation in *Candida* spp. [116]. EO of *Myrtus communis*, a plant typical of the Mediterranean flora, demonstrated an effective inhibition of the adhesion activity, and the biofilm formation in three species isolated from clinical samples: *C. albicans*, *C. parapsilosis* and in a little bit less extent, *C. tropicalis* [117]. The EO of *Mentha suaveolens* plants, collected in various regions of Morocco, showed high percentage of monoterpenes oxides, such as piperitenone oxide and piperitoneoxide, terpenic alcohol (phenol, *p*-cymen-8-ol, geraniol, terpineol, and borneol) and terpenic ketones (pulegone and piperitenone), accounting for 65% to 90% of the total essential oil. This oil was capable of decreasing *C. albicans* adherence (40%) and metabolic activity (70–80%). The inhibition of biofilm formation was also confirmed by SEM observations. EO of *M. suaveolens* also induced morphological surface and cycle modifications of the fungal cell wall; in some cases, cells were swollen and clusters were formed. Investigations performed by confocal laser scanning microscopy (CLSM) and transmission electronic microscopy (TEM), confirmed that EO of *M. suaveolens* was also efficient to induce both the inhibition of the correct synthesis of the cell wall and rarefy the cytoplasmic matrix, with some small electron-transparent vacuoles, and to slow down the yeast cell growth, mainly by reducing the Phase S and condensing the chromatin. Probably, the EO monoterpenes, after their passage across the cell wall, cause damage of the lipid bilayer of the cell membrane and increase the *Candida* cell membrane’s permeability. Their activity is greater when also synthetic drugs are used, since their action leads to the cell damage, by blocking the biofilm [108]. The adverse effects of eukaryotic antimicrobial therapies associated with an increase in resistance to the compounds presently available have boosted research in improving the therapeutic armoury against candidiasis with a newer and cheaper generation of drugs. A novel nerolidol-rich essential oil (EO) derived from *Piper claussenianum* was tested on the growth, transition, formation and stability of biofilms produced by *C. albicans* [118]. Both inflorescence and leaf EOs were evaluated and revealed MIC values ranging from 0.04 to 0.1% and 0.2 to 1.26%, respectively. Leaf EO down-regulates the yeast-to-hyphae transition by 81%, and reduced biofilm formation by about 30 and 50% after incubation for 24 and 48 h, respectively. The EO was also able to reduce the viability of pre-formed biofilm by 63.9%. The simultaneous use of the leaf EO with the synthetic molecule fluconazole revealed a remarkable synergistic effect. Taken together, these results demonstrate that this novel compound represent a promising agent and could reinforce the arsenal of therapeutic alternatives for the treatment of candidiasis, also by negatively influencing the formation and stability of biofilm. Recently, Liu et al. [119] studied the activity of formyl-phloroglucinol meroterpenoids, and in particular, eucarobustol E, which at 16 μg/mL, was capable to block the yeast-to-hypha transition and to reduce the cellular surface hydrophobicity of the *Candida* biofilm cells; this is of particular worth, because relative hydrophobicity is considered as an important pathogenic factor linked to *Candida* adhesion. The action of the molecule gave rise to a marked reduction of the expressions of some genes involved in the growth of the hypha (EFG1, CPH1, TEC1, EED1, UME6, HGC1) and of the ergosterol biosynthesis; on the contrary, other genes involved in the farnesol synthesis were upregulated. EOs can play a relevant role also during the mixed infections due to the concurrent presence of bacteria and fungi. For example, mixed polymicrobial infections due to the presence of bacteria and pathogenic fungi, commonly found in patients with chronic infections, constitute a noteworthy health care burden. *Citrus* EOs are capable of preventing the polymicrobial biofilm formed by *Pseudomonas aeruginosa* and pathogenic fungi, in particular *A. fumigatus* and *Scedosporium apiospermum*, at concentration between 50 mg/L and 250 mg/L [120].These EOs showed a potential of inhibiting fungal growth with MIC in concentrations of 50 mg/L and 250 mg/L. Furthermore, *Citrus* EOs impeded formation of bacterial and fungal monomicrobial biofilms in concentrations of 50 mg/L. The concentration of 10 mg/L EOs inhibited mixed biofilm formation. *Citrus* EOs affected quorum sensing in *P. aeruginosa* and caused fast permeabilisation of *C. albicans* membrane. The EO and the major components of *O. basilicum* var. Maria Bonita, a genetically improved cultivar, were effective against the biofilm formation by two resistant strains of *C. allbicans* and *C. neoformans*. Among its components, geraniol showed better activity than the EO; furthermore, the combination of EO, linalool, or geraniol with the synthetic antifungal fluconazole enhanced their antifungal activity and sub-inhibitory concentrations of the substances resulted in a reduction of the amount of sterol extracted as well as the capsule size, suggesting that they play an important role, in particular by causing the cell wall destruction of *C. neoformans* and membrane irregularities, the presence of vesicles and cell wall thickening in *C. albicans* [121].

## 4. Essential Oils, Quorum Sensing and Mycotoxins

The so-called “quorum sensing” (QS) events are those in which microbial actions or responses are managed by cell density. Secreted signalling molecules, which accumulation can be considered as an extent of cell density [122,123], usually govern such community behaviours. QS has a crucial role in biofilms of all kinds of microorganisms, including fungi [124,125]. The best studied quorum-sensing molecule in *C. albicans* is an inhibitor of hyphal formation, an exogenous molecule called E, E-farnesol. It inhibits biofilm formation when provided early during adherence [126]. Farnesol also accumulates in supernatants of mature biofilms where stimulation of yeast cell production might stimulate their dispersal. Tyrosol, an alcohol derived from tyrosine, has the opposite activity to farnesol, since it stimulates hyphal formation [127]. The addition of the exogenous tyrosol does not have a measurable effect on overall biofilm development but can partially overcome the inhibition of biofilm formation by exogenous farnesol; the whole inhibition of hyphal formation by such supernatants seems to reflect the dominant activity of farnesol. Several other small molecules are detectable in biofilm supernatants, including phenylethyl alcohol, dodecanol and nerolidol [128]. They can inhibit the formation of hyphae, thus support the biofilm dispersal by promoting the formation of the yeast cell. Kerekes et al. [28] investigated the effect of clarysage, juniper, lemon and marjoram EOs and their major components (in particular α-pinene, limonene, linalool and terpinene-4-ol) on the formation of bacterial and yeast biofilms and on *N*-acylhomoserine lactone (AHL)-derived QS. They observed that lemon EO and limonene did not display inhibitory effect, or this effect was minimal on the production of violacein by *Chromobacterium violaceum*. Juniper and a-pinene as well as clary sage and linalool showed a quantity-dependent effect. The volume of α-pinene used did not affect the halo diameter. Marjoram and terpinene-4-ol had the best anti-QS effect at 2 μL. In all cases, the oils proved to be better QS inhibitors than their major components. *Citrus* EOs affected quorum sensing in *P. aeruginosa* and caused fast permeabilisation of *C. albicans* membrane [129]. EOs and their components can also inhibit the expression of some enzymes considered as key elements in the catabolism of carbohydrates, and the synthesis of mycotoxins. Mycotoxins affect about 4.5 billion people in developing countries and mycotoxicosis is considered as one of the top ten most important health risks [130]. The inhibition of fungal pathogens is nowadays even more difficult; synthetic preserving agents are often used to control crop fungal contamination instead of natural alternatives. However, the overuse of synthetic preservatives results in a subsequent increase in drug resistance by fungi, potential dangerous effects for human health due to the residues found in the food items, and the enhancement of fungal consortia [131,132,133]. The economic loss and health hazard caused by the toxigenic fungal contamination is an increasingly severe problem. Approximately, toxigenic fungi contaminate around 25% of cereals grown and then consumed around the world [134]. The contamination causes two problems; on the one hand, leads to the quality deterioration of cereals, and on the other hand, direct infection of immunosuppressed patients is possible. Moreover, mycotoxins are included among the most potent naturally occurring toxic and hepatic carcinogenic compounds, classified as group 1 human carcinogen by the International Agency for Research on Cancer [130]. Some deoxyglucosides of carvacrol, thymol, menthol, fenchol, borneol, S-(–)-perillyl alcohol and citronellol generally demonstrated a noticeable inhibition in the production of mycotoxins, such as ochratoxin and aflatoxin-B2 [135]. Hu et al. [17] found that turmeric EO inhibited the aflatoxin biosynthetic gene expression in *A. flavus*, mainly by acting on two regulatory genes, aflR and aflS, and secondarily on the structural genes aflD, aflM, aflO, aflP and aflQ. This activity leads to a down-regulation of the transcription level of aflatoxin genes, and a consequent reduction of the production of that mycotoxin by the mould. Bruns et al. [80] found an increased production of the mycotoxin gliotoxin, which might be a putative factor of the fungus to persist in chronic lung infections. Therefore, specific glyotoxins are formed onto *A. fumigatus* Af293 biofilm, which can be present on human pulmonary epithelial cells. The expression and production of mycotoxins, in some cases linked to the formation of biofilm, can be increased as a result of single environmental factors such as temperature, water activity (aw) and pH and water activity × temperature interactions, for example under suboptimal growth conditions, when intermediate environmental stress is imposed on *A. parasiticus* (afl genes), *Penicillium verrucosum* (ota genes) and *Fusarium culmorum* (tri genes). Expression of the mycotoxin biosynthesis genes was followed exactly by phenotypic mycotoxin production. Eugenol represents also an active compound of many anti-toxinogenic essential oils, since it blocks aflatoxin B1 (AFB1) production. Its action takes place mainly through the down-regulation of aflatoxin cluster genes. In fact, the expression of 19 out of 27 genes of the cluster was almost no more detectable after eugenol exposure, whereas the expression levels of the other genes were 10-fold to 20-fold reduced [136]. These results are in accordance with a recent work of Jahanshiri et al. [137], who showed that eugenol decreased the expression of some of aflatoxins’ (AF) cluster genes in *A. parasiticus*. EOs usually contain some compounds with anti deoxynivalenol (DON) activity. For instance, EOs extracted from cinnamon, clove, oregano, palmarosa and lemongrass could inhibit DON accumulation in *Fusarium*-infected grains [138,139]; some environmental factors, such as temperature and/or water activity, could influence the anti-mycotoxigenic activity of essential oils indeed [140,141]. A recent research shows that *Ocimum sanctum* EO has a prominent antagonistic activity on the growth of *F. graminearum* [142]. Furthermore, *Zataria multiflora* and *Satureja hortensis* EOs, as well as thymol and carvacrol, were capable of decreasing DON production by *F. graminearum* isolated at 84, 89.1, 95 and 86.6%, respectively [143].

## 5. Conclusions

The fungicide and fungistatic activities of the essential oils, the growing literature on their mechanisms of action, along with the knowledge about their traditional and new uses, emphasize the possible applications of these natural substances in many fields, from human medicine to agriculture, from food technology to the reduction of the use of synthetic drugs and additives. Even if more studies are necessary, the use of essential oils complies with the search for natural substances, safe for human and environmental health. Focal areas deserving further detailed studies appear: (1) the evaluation of the possible synergistic effects among the EOs and/or their components and among essential oils and synthetic molecules; (2) the identification of the active components, taking into account the possible existence of chemo- and ecotypes; (3)studies on their possible toxicity; (4) the identification of the molecular targets of their activity; (5) the possibility to build bio-factories to obtain essential oils with desired chemical and biological properties. In pharmaceutical as well as in food chemistry and technology, EOs possess a pivotal role, both as active substances and as additives. The antifungal, antitoxic and antibiofilm properties of the essential oils can serve as a bridge between their traditional uses and their rational utilization in complementary practices.

## Figures and Tables

**Table 1:** Principal effects of essential oils and/or their components on fungi. References are indicated in parenthesis.

Activity	Oils and/or Components	Activity	Oils and/or Components
Antifungal	*Calamintha nepeta* [6] *Cananga odorata* [18] *Cicuta virosa* [23] *Citrus* [24,25] *Commiphora myrrha* [18] *Coriandrum sativum* [18,24] *Curcuma longa* [16] *Cymbopogon nardus* [26] *Eucalyptus* [24,27] *Hedychium spicatum* [18] *Hyssopus officinalis* [13] *Illicium verum* [24] *Lavandula angustifolia* [13,24] *Matricaria ricutita* [24] *Melaleuca alternifolia* [24]	Antifungal	*Melissa officinalis* [13] *Myristica fragrans* [28] *Myrthaceae* [29] *Ocimum basilicum* [13,24] *Origanum* [13,18,24,28] *Pelargonium graveolens* [24,28] *Piper nigrum* [28] *Salvia officinalis* [13] *Syzygium aromaticum* [28] *Thymus vulgaris* [13,24,28] *Saturjeia hortensis* [24] *Viola odorata* [24] carvacrol [30]
Effect on membrane/wall	*Cinnamomum* [31,32,33] *Citrus* [33,34,35] *Coriaria nepalensis* [36] *Coriandrum sativum* [37] *Juniperus communis* [35] *Litsea cubeba* [35] *Melaleuca alternifolia* [33,38] *Mentha piperita* [33] *Ocimum basilicum* [33] *Origanum* [33,35] *Salvia sclarea* [35] *Syzygium_aromaticum* [39] *Thymus* [33,40] anethole [41] benzyl benzoate [42]	Effect on membrane/wall	1,8-cineole [40] carvacrol [40,43,44] cinnamaldehyde [39,45,46,47] *p*-cymene [40] citral [42] citronellal [42] eugenol [39,42] limonene [35] linalool [35,42] linalyl acetate [42] α-pinene [35] α-terpinene [35,38] terpinene-4-ol [35] thymol [40,43]
Effect on cell growth and morphology	*Eucalyptus* [27] *Thymus* spp. [40] carvacrol [40,43] α-pinene [35,38] 1,8-cineole [40] *p*-cymene [40] citronellal [48] α-terpinene [35,38] γ-terpinene [35,38] terpinene-4-ol [35,38] thymol [40,43]	Inhibition of efflux pump	*Cinnamomum* [31,32,33,39,45,46,49] *Citrus* [33] *Eucalyptus* [33] *Melaleuca alternifolia* [33] *Mentha* [33] *Ocimum basilicum* [33] *Origanum vulgare* [33] *Thymus vulgaris* [33] carvacrol [50] cinnamaldehyde [31,32,39,45,46,49] thymol [50]
Action on fungal mitochondria	*Anethum graveolens* [51] *Artemisia herba alba* [52] *Cananga odorata* [18] *Cinnamomum camphora* [52] *Coriandrum sativum* [18] *Commiphora myrrha* [18] *Hedychium spicatum* [18] *Origanum compactum* [52] *Origanum majorana* [18] lupeol [53] tetraterpenoid [53]	ROS production anti nitric oxide	*Zatharia multiflora* [54] carvacrol [54] *p*-cymene [54] farnesol [55] thymol [54,56]
Synergistic/antagonistic	*Citrus* [25,57] *Coriandrum sativum* [57] *Cymbopogon nardus* [26] *Eucalyptus* [27,57] *Illicium verum* [57] *Lavandula angustifolia* [57] *Matricaria recutita* [57] *Melaleuca alternifolia* [57] *Myrthus* [29] *Ocimum basilicum* [57] *Origanum heracleoticum* [57] *Pelargonium graveolens* [57] *Rosa damascena* [57]	Synergistic/antagonistic	*Satureja hortensis* [57] *Thymus vulgaris* [40,57] *Viola odorata* [57] benzyl benzoate [42] carvacrol [30,40,43,44,58] 1,8-cineole [40] citral [42] citronellal [42] eugenol [42] linalool [42] linalyl acetate [42] thymol [40,42,43]
Inhibition of biofilm development	*Coriandrum sativum* [59] *Croton cajucara* [60,61] *Cymbopogon* [62,63,64,65] *Cytrus* [66] *Eucalyptus* [62] *Laurus nobilis* [67] *Litsea* [35] *Melaleuca alternifolia* [38,68,69] *Mentha* [62,70] *Myrtus communis* [71] *Ocimum* [72,73] *Piper claussenianum* [74] *Rosmarinus officinalis* [75]	Inhibition of biofilm development	*Syzygium aromaticum* [62,64] ρ-cymene [38] *p*-cymene [69] 1-8-cineole [38,69] linalool [60,76] terpinen-4-ol [38,69] terpinolene [38,69] α-terpineol [38,69] eucarobustol E [76,77] eugenol [78] α-terpinene [38,69] γ-terpinene [38,69]
Anti quorum sensing	*Citrus* [35,79] *Juniperus communis* [35] *Mentha piperita* [80] *Origanum* [35] *Salvia sclarea* [35] limonene [35] linalool [35] α-pinene [35] terpinene-4-ol [35]	Effect on micotoxins synthesis/production	*Cinnamomum* [81,82] *Origanum vulgare* [81] *Cymbopogon* [81,82] *Cider* [82] *Citrus* [82] *Eucalyptus* [82] *Mentha* [82] *Ocimum sanctum* [83] *Rosmarinus officinalis* [82] *Satureja hortensis* [84] *Thymus* [82] *Zataria multiflora* [84] 2,3-dideoxyglucosides [85] eugenol [86,87]

## References

[1] Macwan S.R., Dabhi B.K., Aparnathi K.D., Prajapati J.B. (2016). Essential oils of herbs and spices: Their antimicrobial activity and application in preservation of foods. Int. J. Curr. Microbiol. Appl. Sci..

[2] Masango P. (2005). Cleaner production of essential oils by steam distillation. J. Clean. Prod..

[3] Tongnuanchan P., Benjakul S. (2014). Essential oils: Extraction, bioactivities, and their uses for food preservation. J. Food Sci..

[4] Bakkali F., Averbeck S., Averbeck D., Idaomar M. (2008). Biological effects of essential oils—A review. Food Chem. Toxicol..

[5] Mijat Božović M., Garzoli S., Sabatino M., Pepi F., Baldisserotto A., Andreotti E., Romagnoli C., Mai A., Manfredini S., Ragno R. (2017). Essential oil extraction, chemical analysis and anti-Candida activity of *Calamintha nepeta* (L.) Savi subsp. *glandulosa* (Req.) Ball-New Approaches. Molecules.

[6] Burt S.A., Reinders R.D. (2003). Antibacterial activity of selected plant essential oils against *Escherichia coli* O157:H7. Lett. Appl. Microbiol..

[7] Fengfeng W., Yamei J., Xueming X., Na Y. (2017). Electrofluidic pretreatment for enhancing essential oil extraction from citrus fruit peel waste. J. Clean. Prod..

[8] Ravindran R., Jaiswal A.K. (2016). Exploitation of food industry waste for high-value products. Trends Biotechnol..

[9] Niu C., Gilbert E.S. (2004). Colorimetric method for identifying plant essential oil components that affect biofilm formation and structure. Appl. Environm. Microbiol..

[10] Hyldgaard M., Mygind T., Rikke L.M. (2012). Essential oils in food preservation: Mode of action, synergies, and interactions with food matrix components. Front. Microbiol..

[11] Lopez-Reyes J.G., Spadaro D., Preile A., Garibaldi A., Gullino M.L. (2013). Efficacy of plant essential oils on postharvest control of rots caused by fungi on different stone fruits in vivo. J. Food Protect..

[12] De Martino L., De Feo V., Nazzaro F. (2009). Chemical composition and in vitro antimicrobial and mutagenic activities of seven *Lamiaceae* essential oils. Molecules.

[13] Nazzaro F., Fratianni F., De Martino L., Coppola R., De Feo V. (2013). Effects of essential oils on pathogenic bacteria. Pharmaceuticals.

[14] Elshafie H.S., Mancini E., Camele I., De Martino L., De Feo V. (2015). In vivo antifungal activity of two essential oils from Mediterranean plants against postharvest brown rot disease of peach fruit. Ind. Crops Prod..

[15] Hu Y., Zhang J., Kong W., Zhao G., Yang M. (2007). Mechanisms of antifungal and anti-aflatoxigenic properties of essential oil derived from turmeric (*Curcuma longa* L.) on *Aspergillus flavus*. Food Chem..

[16] Kalemba D., Kunicka A. (2003). Antibacterial and antifungal properties of essential oils. Curr. Med. Chem..

[17] Prakash B., Singh P., Kedia A., Dubey N.K. (2012). Assessment of some essential oils as food preservatives based on antifungal, antiaflatoxin, antioxidant, activities and in vivo efficacy in food system. Food Res. Int..

[18] Ghalem B.R., Choudari D.K., Varma A., Tuteja N. (2016). Essential oils as antimicrobial agents against some important plant pathogenic bacteria and fungi. Plant-Microbe Interaction: An Approach to Sustainable Agriculture.

[19] Lang G., Buchbauer G. (2012). A review on recent research results (2008–2010) on essential oils as antimicrobials and antifungals. A review. Flavour Fragr. J..

[20] Fratianni F., De Martino L., Melone A., De Feo V., Coppola R., Nazzaro F. (2010). Preservation of chicken breast meat treated with thyme and balm essential oils. J. Food Sci..

[21] Edris A.E. (2007). Pharmaceutical and therapeutic potentials of essential oils and their individual volatile constituents: A review. Phytother. Res..

[22] Tian J., Ban B., Zeng H., He J., Bo H., Wang Y. (2011). Chemical composition and antifungal activity of essential oil from *Cicuta virosa* L. var. *latisecta* Celak. Int. J. Food Microbiol..

[23] Bajpai V.K., Kang S., Xu H., Lee S.G., Baek K.H., Kang S.-C. (2011). Potential roles of essential oils on controlling plant pathoenic bacteria *Xanthomonas* species: A review. Plant Pathol. J..

[24] Wu X.Z., Cheng A.X., Sun L.M., Lou H.X. (2008). Effect of plagiochin E, an antifungal macrocyclic bis (bibenzyl), on cell wall chitin synthesis in *Candida albicans*. Acta Pharmacol. Sin..

[25] Yutani M., Hashimoto Y., Ogita A., Kubo I., Tanaka T., Fujita K. (2011). Morphological changes of the filamentous fungus *Mucor mucedo* and inhibition of chitin synthase activity induced by anethole. Phytother. Res..

[26] Gogoi P., Baruah P., Nath S.C. (2008). Microbiological Research. Effects of *Citrus sinensis* (L.) Osbeck epicarp essential oil on growth and morphogenesis of *Aspergillus niger* (L.) Van Tieghem.). Microbiol. Res..

[27] Iscan G., Iscan A., Demirci F. (2016). Anticandidal effects of thymoquinone: Mode of action determined by transmission electron microscopy (TEM). Nat. Prod. Commun..

[28] Hammer K.A., Carson C.F., Riley T.V. (2004). Antifungal effects of *Melaleuca alternifolia* (tea tree) oil and its components on *Candida albicans*, *Candida glabrata* and *Saccharomyces cerevisiae*. J. Antimicrob. Chemother..

[29] Kerekes E.B., Deak E., Tako M., Tserennadmid R., Petkovits T., Vagvolgyi C., Krisch J. (2013). Anti-Bio film forming and anti-quorum sensing activity of selected essential oils and their main components on food-related microorganisms. J. Appl. Microbiol..

[30] Rajput S.B., Karuppayil S.M. (2013). Small molecules inhibit growth, viability and ergosterol biosynthesis in *Candida albicans*. Springerplus.

[31] Ahmad A., Khan A., Kumar P., Bhatt R.P., Manzoor N. (2011). Antifungal activity of *Coriaria nepalensis* essential oil by disrupting ergosterol biosynthesis and membrane integrity against *Candida*. Yeast.

[32] Freires de Almeida I., Murata R.M., Furletti V.F., Sartoratto A., Matias de Alencar S., Figueira G.M., de Oliveira Rodrigues J.A., Duarte M.C.T., Rosalen P.L. (2014). *Coriandrum sativum* L. (Coriander) essential oil: Antifungal activity and mode of action on *Candida* spp. and molecular targets affected in human whole-genome expression. PLoS ONE.

[33] Chen Y., Zeng H., Tian J., Ban X., Ma B., Wang Y. (2013). Antifungal mechanism of essential oil from *Anethum graveolens* seeds against *Candida albicans*. J. Med. Microbiol..

[34] Bakkali F., Averbeck S., Averbeck D., Zhiri A., Baudouxc D., Idaomar M. (2006). Antigenotoxic effects of three essential oils in diploid yeast (*Saccharomyces cerevisiae*) after treatments with UVC radiation, 8-MOP plus UVA and MMS. Mutat. Res..

[35] Haque E., Irfan S., Kamil M., Sheikh S., Hasan A., Ahmad A., Lakshmi V., Nazir A., Mir S.S. (2016). Terpenoids with antifungal activity trigger mitochondrial dysfunction in *Saccharomyces cerevisiae*. Microbiology.

[36] Set-Young D., Monk B.C., Mason A.B., Perlin D.S. (1997). Exploring an antifungal target in the plasma membrane H^+^ ATPase of fungi. Biochim. Biophys. Acta.

[37] Perlin D.S., Seto-Young D., Monk B.C. (2006). The plasma membrane H^+^ ATPase of fungi. A candidate drug target?. Ann. N. Y. Acad. Sci..

[38] Ahmad A., Khan A., Manzoor N. (2013). Reversal of efflux mediated antifungal resistance underlies synergistic activity of two monoterpenes with fluconazole. Eur. J. Pharm. Sci..

[39] Belenky P., Collins J.J. (2011). Antioxidant strategies to tolerate antibiotics. Science.

[40] Gusarov I., Shatalin K., Starodubtseva M., Nudler E. (2009). Endogenous nitric oxide protects bacteria against a wide spectrum of antibiotics. Science.

[41] Shen Q., Zhou W., Li H., Hu L., Mo H. (2016). ROS involves the fungicidal actions of thymol against spores of Aspergillus flavus via the induction of nitric oxide. PLoS ONE.

[42] Vieira A.L., Linares E., Augusto O., Gomes S.L. (2009). Evidence of a Ca^2+^-NO-cGMP signaling pathway controlling zoospore biogenesis in the aquatic fungus *Blastocladiella emersonii*. Fungal Genet. Biol..

[43] Kong W., Huang C., Chen Q., Zou Y., Zhang J. (2012). Nitric oxide alleviates heat stress-induced oxidative damage in *Pleurotus eryngii* var. tuoliensis. Fungal Genet. Biol..

[44] Kohanski M.A., Dwyer D.J., Hayete B., Lawrence C.A., Collins J.J. (2007). A common mechanism of cellular death induced by bactericidal antibiotics. Cell.

[45] Cotoras M., Castro P., Vivanco H., Melo R., Mendoza L. (2013). Farnesol induces apoptosis-like phenotype in the phytopathogenic fungus *Botrytis cinerea*. Mycologia.

[46] Kavoosi G., Teixeira da Silva J.A., Saharkhiz M.J. (2012). Inhibitory effects of *Zataria multiflora* essential oil and its main components on nitric oxide and hydrogen peroxide production in lipopolysaccharide-stimulated macrophages. J. Pharm. Pharmacol..

[47] Adams R.P. (2001). Identification of essential oil components by gas chromatography-mass spectroscopy. J. Am. Soc. Mass Spectrom..

[48] Stevic T., Beric T., Savikin K., Sokovic M., GoCevac D., Dimkic I., Stankovic S. (2014). Antifungal activity of selected essential oils against fungi isolated from medicinal plant. Ind. Crops Prod..

[49] Lima I.O., de Oliveira Pereira F., de Oliveira W.A., de Oliveira Lima E., Menezes E.A., Cunha F.A., de Fátima M.F.M.D. (2013). Antifungal activity and mode of action of carvacrol against *Candida albicans* strains. J. Essent. Oil Res..

[50] Dorman H.J.D., Deans S.G. (2000). Antimicrobial agents from plants: Antibacterial activity of plant volatile oils. J. Appl. Microbiol..

[51] Ultee A., Bennik M.H., Moezelaar R. (2002). The phenolic hydroxyl group of carvacrol is essential for action against the food-borne pathogen *Bacillus cereus*. Appl. Environ. Microbiol..

[52] Pina-Vaz C., Rodrigues A.G., Pinto E., Costa-De-Oliveira S., Tavares C., Satgueiro L., Cavaleiro C., Goncalves M.J., Martinez-De-Oliveira J. (2004). Antifungal activity of *Thymus* oils and their major compounds. J. Eur. Acad. Derm. Venereol..

[53] Chavan P.S., Tupe S.G. (2014). Antifungal activity and mechanism of action of carvacrol and thymol against vineyard and wine spoilage yeasts. Food Contr..

[54] Nobrega R.O., Teixeira A.P., Oliveira W.A., Lima E.O., Lima I.O. (2016). Investigation of the antifungal activity of carvacrol against strains of *Cryptococcus neoformans*. Pharm. Biol..

[55] Carrillo-Hormaza L., Mora C., Alvarez R., Alzate F., Osorio E. (2015). Chemical composition and antibacterial activity against *Enterobacter cloacae* of essential oils from *Asteraceae* species growing in the Paramos of Colombia. Ind. Crops Prod..

[56] Tolba H., Moghrani H., Benelmouffolk A., Kellou D., Maachi R. (2015). Essential oil of Algerian *Eucalyptus citriodora*: Chemical composition, antifungal activity. J. Med. Mycol..

[57] Lee Y.S., Kim J., Shin S.C., Lee S.G., Park I.K. (2008). Antifungal activity of *Myrtaceae* essential oils and their components against three phytopathogenic fungi. Flavour Fragr. J..

[58] Rammanee K., Hongpattarakere T. (2011). Effects of tropical citrus essential oils on growth, aflatoxin production, and ultrastructure alterations of *Aspergillus flavus* and *Aspergillus parasiticus*. Food Process Biotechnol..

[59] Trindade L.A., de Arajio Oliveira J., de Castro R.D., De Oliveira Lima E. (2015). Inhibition of adherence of *C. albicans* to dental implants and cover-screws by *Cymbopogon nardus* essential oil and citronellal. Clin. Oral Investig..

[60] Zore G.B., Thakre A.D., Jadhav S., Karuppayil S.M. (2011). Terpenoids inhibit *Candida albicans* growth by affecting membrane integrity and arrest of cell cycle. Phytomedicine.

[61] Lee H.S., Ahn Y.J. (1998). Growth-inhibiting effects of *Cinnamomum cassia* bark-derived materials on human intestinal bacteria. J. Agric. Food Chem..

[62] Friedman M., Henika P.R., Mandrell R.E. (2002). Bactericidal activities of plant essential oils and some of their isolated constituents against *Campylobacter jejuni*, *Escherichia coli*, *Listeria monocytogenes*, and *Salmonella enterica*. J. Food Prot..

[63] Chang S.T., Chen P.F., Chang S.C. (2001). Antibacterial activity of leaf essential oils and their constituents from *Cinnamomum osmophloeum*. J. Ethnopharmacol..

[64] Shreaz S., Sheikh R.A., Bhatia R., Hashmi A.A., Manzoor N., Khan L.A. (2010). Anticandidal activity of cinnamaldehyde, its ligand and Ni (II) complex: Effect of increase in ring and side chain. Microb. Pathog..

[65] Julnar U., Sawsan K., Pascale B., Yolla B.M., Hania N.C. (2003). Comparative study on the effect of cinnamon and clove extracts and their main components on different types of ATPases. Hum. Exp. Toxicol..

[66] Bang K.H., Lee D.W., Park H.M., Rhee Y.H. (2000). Inhibition of fungal cell wall synthesizing enzymes by trans-cinnamaldehyde. Biosci. Biotechnol. Biochem..

[67] Xie X.M., Fang J.R., Xu Y. (2004). Study of antifungal effect of cinnamaldehyde and citral on *Aspergillus flavus*. Food Sci..

[68] Hossain F., Follett P., Vu K.D., Harich M., Salmieri S., Lacroix M. (2016). Evidence for synergistic activity of plant-derived essential oils against fungal pathogens of food. Food Microbiol..

[69] Suppakul P., Miltz J., Sonneveld K., Bigger S.W. (2003). Active packaging technologies with an emphasis on antimicrobial packaging and its applications. J. Food Sci..

[70] Bound J.D., Murthy P.S., Srinivas P. (2016). 2,3-Dideoxyglucosides of selected terpene phenols and alcohols as potent antifungal compounds. Food Chem..

[71] Wu Y., Yang Q.O., Tao N. (2016). Plasma membrane damage contributes to antifungal activity of citronellal against *Penicillium digitatum*. J. Food Sci. Technol..

[72] Lynch A.S., Robertson G.T. (2008). Bacterial and fungal biofilm infections. Annu. Rev. Med..

[73] Costa-Orlandi C.B., Sardi J.C.O., Pitangui N.S., de Oliveir H.C., Scorzoni L., Galeane M.C., Medina-Alarcón K.P., Melo W.C.M.A., Marcelino M.Y., Braz J.D. (2017). Fungal biofilms and polymicrobial diseases. J. Fungi.

[74] Costerton J.W., Stewart P.S., Greenberg E.P. (1999). Bacterial biofilms: A common cause of persistent infections. Science.

[75] Sardi J.C., Pitangui N.S., Rodriguez-Arellanes G., Taylor M.L., Fusco-Almeida A.M., Mendes-Giannini M.J. (2014). Highlights in pathogenic fungal biofilms. Rev. Iberoam. Micol..

[76] Blankenship J.R., Mitchell A.P. (2006). How to build a biofilm: A fungal perspective. Curr. Opin. Microbiol..

[77] Fanning S., Mitchell A.P. (2012). Fungal biofilms. PLoS Pathog..

[78] Martinez L.R., Fries B.C. (2010). Fungal biofilms: Relevance in the setting of human disease. Curr. Fungal Infect. Rep..

[79] Beauvais A., Muller F.M., Latge J.P., Steinbach W.J. (2009). Biofilm formation in Aspergillus fumigatus. Aspergillus Fumigatus and Aspergillosis.

[80] Husain F.M., Ahmad I., Khan M.S., Ahmad E., Tahseen O., Khan M.S., Alshabib N.A. (2015). Sub-MICs of *Mentha piperita* essential oil and menthol inhibits AHL mediated quorum sensing and biofilm of Gram-negative bacteria. Front Microbiol..

[81] LaFleur M.D., Kumamoto C.A., Lewis K. (2006). *Candida albicans* biofilms produce antifungal-tolerant persister cells. Antimicrob. Agents Chemother..

[82] Ramage G., Mowat E., Jones B., Williams C., Lopez-Ribot J. (2009). Our current understanding of fungal biofilms. Crit. Rev. Microbiol..

[83] Henriques M., Azeredo J., Oliveira R. (2006). *Candida albicans* and *Candida dubliniensis*: Comparison of biofilm formation in terms of biomass and activity. Br. J. Biomed. Sci..

[84] Paramonova E., Krom B.P., van der Mei H.C., Busscher H.J., Sharma P.K. (2009). Hyphal content determines the compression strength of *Candida albicans* biofilms. Microbiology.

[85] Di Bonaventura G., Pompilio A., Picciani C., Iezzi M., D’Antonio D., Piccolomini R. (2006). Biofilm formation by the emerging fungal pathogen *Trichosporon asahii*: Development, architecture, and antifungal resistance. Antimicrob. Agents Chemother..

[86] Davis L.E., Cook G., Costerton J.W. (2002). Biofilm on ventriculo-peritoneal shunt tubing as a cause of treatment failure in coccidioidal meningitis. Emerg. Infect. Dis..

[87] Martinez L.R., Casadevall A. (2007). *Cryptococcus neoformans* biofilm formation depends on surface support and carbon source and reduces fungal cell susceptibility to heat, cold, and UV light. Appl. Environ. Microbiol..

[88] Freisesleben S.H., Jager A.K. (2014). Correlation between plant secondary metabolites and their antifungal mechanism—a review. Med. Arom. Plants.

[89] Harding M.W., Marques L.L., Howard R.J., Olson M.E. (2009). Can filamentous fungi form biofilms?. Trends Microbiol..

[90] Silva S., Henriques M., Martins A., Oliveira R., Williams D., Azeredo J. (2009). Biofilms of non-*Candida albicans Candida* species: Quantification, structure and matrix composition. Med. Mycol..

[91] Gibbons J.G., Beauvais A., Beau R., McGary K.L., Latge J.P., Rokas A. (2011). Global transcriptome changes underlying colony growth in the opportunistic human pathogen *Aspergillus fumigatus*. Eukaryot. Cell.

[92] Sahni N., Yi S., Daniels K.J., Srikantha T., Pujol C., Soll D.R. (2009). Genes selectively up-regulated by pheromone in white cells are involved in biofilm formation in *Candida albicans*. PLoS Pathog..

[93] Yi S., Sahni N., Daniels K.J., Lu K.L., Srikantha T., Huang G., Garnaas A.M., David R., Soll D.R. (2011). Alternative mating type configurations (a/alpha versus *a/a* or alpha/alpha) of *Candida albicans* result in alternative biofilms regulated by different pathways. PLoS Biol..

[94] Ramage G., Saville S.P., Thomas D.P., Lopez-Ribot J.L. (2005). *Candida* biofilms: An update. Eukaryot. Cell.

[95] Sheehan D.J., Hitchcock C.A., Sibley C.M. (1999). Current and emerging azole antifungal agents. Clin. Microbiol. Rev..

[96] Bruzual I., Riggle P., Hadley S., Kumamoto C.A. (2007). Biofilm formation by fluconazole-resistant *Candida albicans* strains is inhibited by fluconazole. J. Antimicrob. Chemother..

[97] Meyer B. (2003). Approaches to prevention, removal and killing of biofilms. Int. Biodeter. Biodegr..

[98] Sanglard D., Ischer F., Parkinson T., Falconer D., Bille J. (2003). *Candida albicans* mutations in the ergosterol biosynthetic pathway and resistance to several antifungal agents. Antimicrob. Agents Chemother..

[99] Vishnu Agarwal V., Lal P., Pruthi V. (2010). Effect of plant oils on *Candida albicans*. J. Microbiol. Immunol. Infect..

[100] Carson C.F., Hammer K.A., Riley T.V. (2006). *Melaleuca alternifolia* (tea tree) oil: A review of antimicrobial and other medicinal properties. Clin. Microbiol. Rev..

[101] Ramage G., Milligan S., Lappin D.F., Sherry L., Sweeney P., Williams C., Bagg J., Culshaw S. (2012). Anti-fungal, cytotoxic, and immunomodulatory properties of tea tree oil and its derivative components: Potential role in management of oral candidosis in cancer patients. Front. Microbiol..

[102] Furletti V.F., Teixeira I.P., Obando-Pereda G., Mardegan R.C., Sartoratto A., Figueira G.M., Duarte R.M., Rehder V.L., Duarte M.C., Höfling J.F. (2011). Action of *Coriandrum sativum* L. essential oil upon oral *Candida albicans* biofilm formation. Evid. Based Complement. Altern. Med..

[103] Thaweboon S., Thaweboon B. (2009). In vitro antimicrobial activity of *Ocimum americanum* L. essential oil against oral microorganisms. Southeast Asian J. Trop. Med. Public Health.

[104] Hsu C.C., Lai W.L., Chuang K.C., Lee M.H., Tsai Y.C. (2013). The inhibitory activity of linalool against the filamentous growth and biofilm formation in *Candida albicans*. Med. Mycol..

[105] Alviano W.S., Mendonca-Filho R.R., Alviano D.S., Bizzo H.R., Souto-Padron T., Rodrigues M.L., Bolognese A.M., Alviano C.S., Souza M.M.G. (2005). Antimicrobial activity of *Croton cajucara* Benth linalool-rich essential oil on artificial biofilms and planktonic microorganisms. Oral Microbiol. Immunol..

[106] Rukayadi Y., Hwang J.K. (2013). In vitro activity of xanthorrhizol isolated from the rhizome of Javanese turmeric (*Curcuma xanthorrhiza* Roxb.) against *Candida albicans* biofilms. Phytother. Res..

[107] He M., Du M., Fan M., Bian Z. (2007). In vitro activity of eugenol against *Candida albicans* biofilms. Mycopathologia.

[108] Agarwal V., Lal P., Pruthi V. (2008). Prevention of *Candida albicans* biofilm by plant oils. Mycopathologia.

[109] Stringaro A., Vavala E., Colone M., Pepi F., Mignogna G., Garzoli S., Cecchetti S., Ragno R., Angiolella L. (2014). Effects of *Mentha suaveolens* essential oil alone or in combination with other drugs in *Candida albicans*. Evid. Based Complement. Altern. Med..

[110] Tyagi A.K., Malik A. (2010). Liquid and vapour-phase antifungal activities of selected essential oils against *Candida albicans*: Microscopic observations and chemical characterization of *Cymbopogon citratus*. BMC Complement. Altern. Med..

[111] Chifiriuc C., Grumezescu V., Grumezescu A.M., Saviuc C., Lazăr V., Andronescu E. (2012). Hybrid magnetite nanoparticles/*Rosmarinus officinalis* essential oil nanobiosystem with antibiofilm activity. Nanosc. Res. Lett..

[112] Mironescu M., Mironescu I.D., Georgescu C. (2010). Microstructural changes induced by five new biocidal formulations on moulds. Ann. Rom. Soc. Cell Biol..

[113] Khan M.S., Ahmad I. (2012). Biofilm inhibition by *Cymbopogon citratus* and *Syzygium aromaticum* essential oils in the strains of *Candida albicans*. J. Ethnopharmacol..

[114] De Toledo L.G., Dos Santos Ramos M.A., Spósito L., Castilho E.M., Pavan F.R., De Oliveira Lopes E., Zocolo G.J., Silva F.A.N., Soares T.H., dos Santos A.G. (2016). Essential oil of *Cymbopogon nardus* (L.) Rendle: A strategy to combat fungal infections caused by *Candida* species. Int. J. Mol. Sci..

[115] Caputo L., Nazzaro F., Souza L., De Martino L., Fratianni F., Coppola R., De Feo V. (2017). *Laurus nobilis*: Composition of essential oil and its biological activities. Molecules.

[116] Peixoto L.R., Rosalen P.L., Silva Ferreira G.L., Freires I.A., Galbiatti de Carvalho F., Castellano L.R., Dias de Castro R. (2017). Antifungal activity, mode of action and anti-biofilm effects of *Laurus nobilis Linnaeus* essential oil against *Candida* spp.. Arch. Oral Biol..

[117] Rane H.S., Bernardo S.M., Howell A.B., Lee S.A. (2013). Cranberry-derived proanthocyanidins prevent formation of *Candida albicans* biofilms in artificial urine through biofilm- and adherence-specific mechanisms. J. Antimicrob. Chemother..

[118] Cannas S., Molicotti P., Usai D., Maxia A., Zanetti S. (2014). Antifungal, anti-biofilm and adhesion activity of the essential oil of *Myrtus communis* L. against *Candida* species. Nat. Prod. Res..

[119] Curvelo J.A.R., Marques A.M., Barreto A.L.S., Romanos M.T.V., Portela M.B., Kaplan M.A.C., Soares R.M.A. (2014). A novel nerolidol-rich essential oil from *Piper claussenianum* modulates *Candida albicans* biofilm. J. Med. Microbiol..

[120] Liu R.H., Shang Z.C., Yang M.H., Kong L.Y. (2017). In vitro antibiofilm activity of Eucarobustol E against *Candida albicans*. Antimicrob. Agents Chemother..

[121] Pekmezovic M., Aleksic I., Barac A., Arsic-Arsenijevic V., Vasiljevic B., Nikodinovic-Runic J., Senerovic L. (2016). Prevention of polymicrobial biofilms composed of *Pseudomonas aeruginosa* and pathogenic fungi by essential oils from selected *Citrus* species. Pathog. Dis..

[122] Cardoso N.N.R., Alviano C.S., Blank A.F., Romanos M.T.V., Fonseca B.B., Rozental S., Rodrigues I.A., Alviano D.S. (2016). Synergism effect of the essential oil from *Ocimum basilicum* var. Maria Bonita and its major components with fluconazole and its influence on ergosterol biosynthesis. Evid. Based Complemt. Altern. Med..

[123] Keller L., Surette M.G. (2006). Communication in bacteria: An ecological and evolutionary perspective. Nat. Rev. Microbiol..

[124] Nazzaro F., Fratianni F., Coppola R. (2013). Quorum sensing and phytochemicals. Int. J. Mol. Sci..

[125] Siehnel R., Traxler B., An D.D., Parsek M.R., Schaefer A.L., Singh P.K. (2010). A unique regulator controls the activation threshold of quorum-regulated genes in *Pseudomonas aeruginosa*. Proc. Natl. Acad. Sci. USA.

[126] Peleg A.Y., Hogan D.A., Mylonakis E. (2010). Medically important bacterial-fungal interactions. Nat. Rev. Microbiol..

[127] Ramage G., Saville S.P., Wickes B.L., Lopez-Ribot J.L. (2002). Inhibition of *Candida albicans* biofilm formation by farnesol, a quorum-sensing molecule. Appl. Environ. Microbiol..

[128] Alem M.A., Oteef M.D., Flowers T.H., Douglas L.J. (2006). Production of tyrosol by *Candida albicans* biofilms and its role in quorum sensing and biofilm development. Eukariot. Cell.

[129] Martins M., Henriques M., Azeredo J., Rocha S.M., Coimbra M.A., Oliveira R. (2007). Morphogenesis control in *Candida albicans* and *Candida dubliniensis* through signaling molecules produced by planktonic and biofilm cells. Eukariot. Cell.

[130] Singh P., Shukla R., Prakash B., Kumar A., Singh S., Mishra P.K., Dubey N.K. (2010). Chemical profile, antifungal, antiaflatoxigenic and antioxidant activity of *Citrus maxima* Burm. and *Citrus sinensis* (L.) Osbeck essential oils and their cyclic monoterpene, DL-limonene. Food Chem. Toxicol..

[131] Williams J.H., Phillips T.D., Jolly P.E., Stiles J.K., Jolly C.M., Aggarwal D. (2004). Human aflatoxicosis in developing countries: A review of toxicology, exposure, potential health consequences, and interventions. Am. J. Clin. Nutr..

[132] White T.C., Marr K.A., Bowden R.A. (1998). Clinical, cellular, and molecular factors that contribute to antifungal drug resistance. Clin. Microbiol. Rev..

[133] Brul S., Coote P. (1999). Preservative agents in foods: Mode of action and microbial resistance mechanisms. Int. J. Food Microbiol..

[134] Hurtado-McCormick S., Sánchez L., Martínez J., Calderón C., Calvo D., Narváez D., Lemus M., Groot H., Susa M.R. (2016). Fungi in biofilms of a drinking water network: Occurrence, diversity and mycotoxins approach. Water Sci. Technol..

[135] Dvegowda G., Raju M.V.L.N., Swamy H.V.L.N. (1998). Mycotoxins: Novel solutions for their counteraction. Feedstuffs.

[136] Bruns S., Seidler M., Albrecht D., Salvenmoser S., Remme N., Hertweck C., Brakhage A.A., Kniemeyer F.M. (2010). Functional genomic profiling of *Aspergillus fumigatus* biofilm reveals enhanced production of the mycotoxin gliotoxin. Proteomics.

[137] Caceres I., El Khoury R., Medina A., Lippi Y., Naylies C., Atoui A., El Khoury A., Oswald I.P., Bailly J.D., Puel O. (2016). Deciphering the anti-aflatoxinogenic properties of eugenol using a large-scale q-PCR approach. Toxins.

[138] Jahanshiri Z., Shams-Ghahfarokhi M., Allameh A., Razzaghi-Abyaneh M. (2015). Inhibitory effect of eugenol on aflatoxin B1 production in *Aspergillus parasiticus* by downregulating the expression of major genes in the toxin biosynthetic pathway. World J. Microbiol. Biotechnol..

[139] Marín S., Velluti A., Ramos A.J., Sanchis V. (2004). Effect of essential oils on zearalenone and deoxynivalenol production by *Fusarium graminearum* in non-sterilized maize grain. Food Microbiol..

[140] Perczak A., Juś K., Marchwińska K., Gwiazdowska D., Waśkiewicz A., Goliński P. (2016). Degradation of zearalenone by essential oils under in vitro conditions. Front. Microbiol..

[141] Velluti A., Sanchis V., Ramos A.J., Turon C., Marín S. (2004). Impact of essential oils on growth rate, zearalenone and deoxynivalenol production by *Fusarium graminearum* under different temperature and water activity conditions in maize grain. J. Appl. Microbiol..

[142] Kalagatur N.K., Mudili V., Siddaiah C., Gupta V.K., Natarajan G., Sreepathi M.H., Vardhan B.H., Putcha V.L.R. (2015). Antagonistic activity of *Ocimum sanctum* L. essential oil on growth and zearalenone production by *Fusarium graminearum* in maize grains. Front. Microbiol..

[143] Lahooji A., Mirabolfathy M., Karami-Osboo R. (2010). Effect of *Zataria multiflora* and *Satureja hortensis* essential oils, thymol and carvacrol on growth of *Fusarium gramineum* isolates and deoxynivalenol production. Iran. J. Plant Pathol..

